# High protein-containing new food by cell powder meat

**DOI:** 10.1038/s41538-023-00191-5

**Published:** 2023-04-11

**Authors:** Bumgyu Choi, Sohyeon Park, Milae Lee, Sungwon Jung, Hyun Lee, Geul Bang, Jiyu Kim, Heeyoun Hwang, Ki Hyun Yoo, Dongoh Han, Seung Tae Lee, Won-Gun Koh, Jinkee Hong

**Affiliations:** 1 Department of Chemical & Biomolecular Engineering, College of Engineering, Yonsei University, 50 Yonsei-ro, Seodaemun-gu, Seoul, 03722 Republic of Korea; 2 Department of Animal Life Science, Kangwon National University, Chuncheon, 24341 Republic of Korea; 3 Research Center for Bioconvergence Analysis, Korea Basic Science Institute, Daejeon, 28119 Republic of Korea; 4 SIMPLE Planet Inc., 48 Achasan-ro 17-gil, Seongdong-gu, Seoul, 04799 Republic of Korea; 5 Department of Applied Animal Science, Kangwon National University, Chuncheon, 24341 Republic of Korea

**Keywords:** Biomaterials - cells, Biomaterials - proteins

## Abstract

Demand for a new protein source to replace meat is increasing to solve various issues such as limited resources and food shortages. Diverse protein sources are being developed, but alternative proteins such as plants or insects need to improve people’s perceptions and organoleptic properties. Therefore, cell-based meat research is intensively conducted, and most studies are aimed at scale-up and cost-down via the research of scaffolds and culture media. Here, we proposed a new food by cell powder meat (CPM), which has a high protein content and a meaty flavor. The powder was manufactured 76% more cost-effectively with less serum than the conventional culture medium and without 3D scaffold. Due to its comprehensive characteristics, the potential applicability of CPM in the cell-based meat industry could be expected.

## Introduction

With the increasing seriousness of global population growth, environmental problems, and limited resources, new approaches are being developed to replace traditional meat in order to address the environmental, and animal welfare issues associated with traditional livestock farming^1^. Hence, there has been increasing interest in meat substitutes as future food, and various protein sources ranging from plant-based meat alternatives to proteins extracted from microorganisms, microalgae, and insects are being developed. In particular, numerous studies are being conducted on cell-based meat (cultured or cultivated meat) as a sustainable food^2,3^.

Cell-based meat is generally produced by extracting cells from livestock, proliferating them in large quantities, allowing them to differentiate on scaffolds, and processing them to make final products. The most important factor in the commercialization of cell-based meat is the scaling up of production^4–6^. Among these aspects, the scaffold is essential for the mass production of cell-based meat as well as forming a part of the food product itself^7,8^. However, unlike the scaffolds used for tissue engineering, only food-grade materials should be used when developing a scaffold for cell-based meat and the production cost needs to be low. In addition, the requirement to obtain food-grade approval from food institutions such as the FDA remains a major obstacle to the commercialization of cell-based meat^9^. Therefore, recently, research using cell sheet technology that does not use a 3D scaffold has been presented^4,7,10^.

**Fig. 1:**
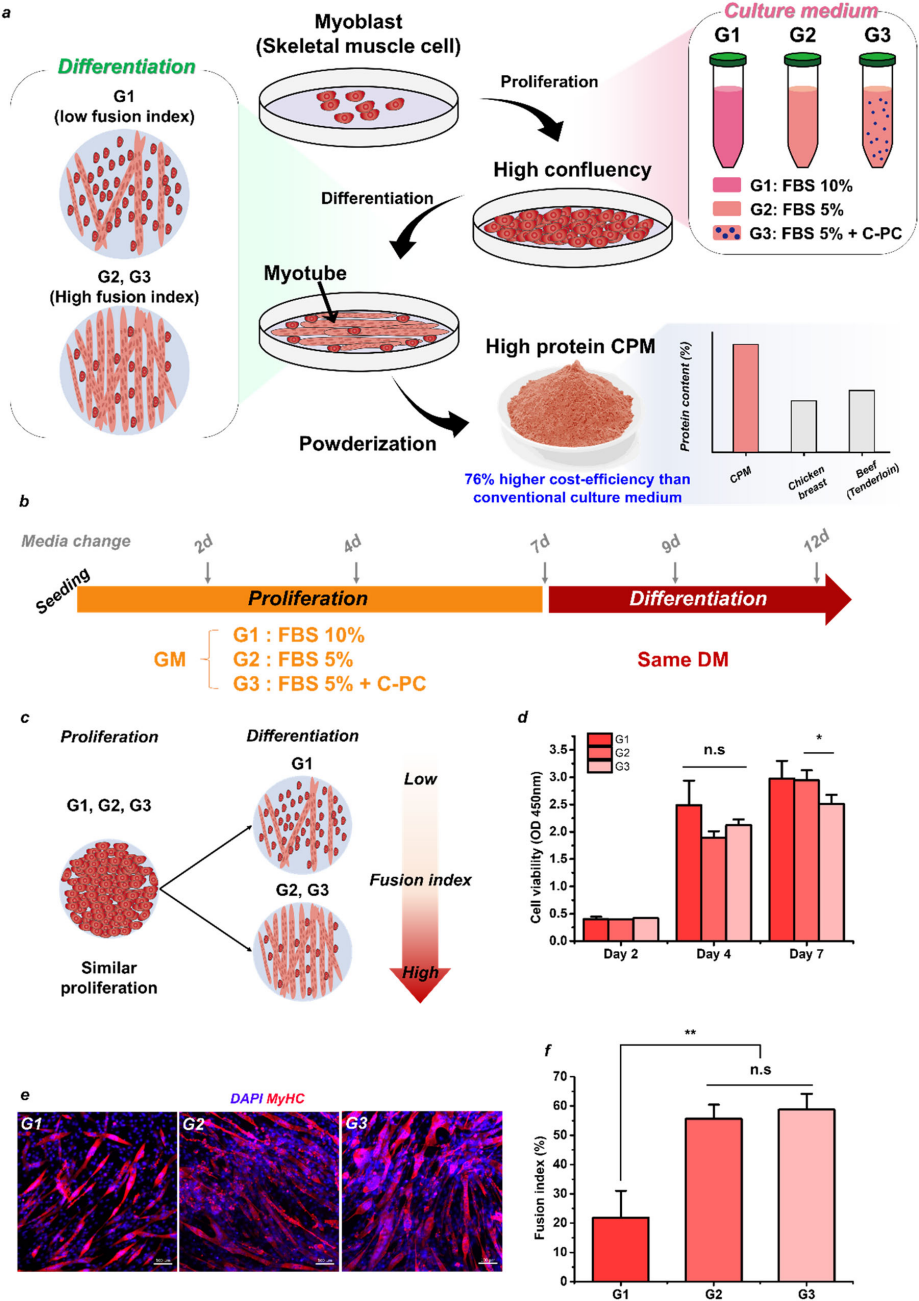
A schematic illustration showing the manufacturing process of cell powder meat (CPM) and cell culture, proliferation, and differentiation in G1, G2, and G3. **a** The manufacturing process of cell powder meat (CPM), the effect of each medium, and the advantages of CPM. Schematic diagrams showing **b**, the conditions (growth media; GM, differentiation media; DM) and process of cell culture and **c**, the anticipated results; **d**, the quantification of cell proliferation via the cck-8 assay; **e** confocal images of the cultured C2C12 cells stained with DAPI (blue) and MyHC (red) (scale bar: 100 μm); **f**, the fusion index of the cultured cells. In parts **d**, **f** the data are presented as the mean ± standard deviation (*n* = 3); for the *t* test, * indicates *p* < 0.05, and ** indicates *p* < 0.01, statistically non-significance (n.s). All experiments were conducted independently.

Using food powder as an example, the present study focuses on the manufacture of cell-based meat without the use of a scaffold, thereby providing high commercialization potential due to excellent price competitiveness and high utility. Food powder is used for various purposes such as flour, salt, and as a colorant, and provides a solution to the complexity of food production in daily life^11^. Food powder has beneficial properties such as physicochemical stability as well as being easy to preserve, transport, and process^12^. Despite the advantages of these food powders, alternative proteins such as plant and insect-based powders have limitations in that consumers’ recognition and sensory characteristics need to be improved, so an enhanced form of alternative protein is required^2^. Thus, the fabrication of cell powder meat (CPM) with a suitable meat flavor and high protein content for use as a meat substitute is described herein as shown in Fig. 1a. Moreover, this high-protein CPM is manufactured using fewer serum-media than traditional culture media, thereby reducing the price. In addition, the as-fabricated CPM presents possibilities in the cell-based meat market due to its high utility and relative ease of manufacture compared to the existing method of using a scaffold. Hence, this process is expected to serve as a cornerstone for making various types of cell-based meat products, ranging from seasonings such as beef powder to cell-based meat that can be kneaded like flour.

## Results and discussions

### C2C12 cell proliferation and differentiation in various media

The C2C12 culture process is shown schematically in Fig. 1b. The control group is G1: FBS 10% and experimental groups are G2: FBS 5%, G3: FBS 5% + C-PC (C-phycocyanin). C-pc is known to be used as a substance that can replace FBS by helping myoblast proliferation^7^. The anticipated results of this procedure are shown schematically in Fig. 1c, and the results of cell proliferation are presented for each group in Fig. 1d. Here, the cells are seen to proliferate most rapidly in G1, which had a high FBS content, during the first 4 days, and perhaps also during the 7-day period, although the difference is not statistically significant. Thus, the degree of cell proliferation before replacing the media with DM is similar in all three groups. In other words, there is no significant difference in the number of cells cultured in G1, G2, and G3, although the number of cells cultured in G1 was slightly higher on average.

The results of further culture in DM for 5 days are revealed by the confocal images of the stained samples in Fig. 1e and Figure S1, where the expression of MyHC verifies the differentiation of the C2C12 myoblasts into myotubes. In contrast to the proliferation behavior indicated in Fig. 1d, the experimental groups (G2 and G3 with low FBS content) exhibit a high fusion index of 55.6% and 58.8%, respectively, while the control group (G1 with high FBS content) exhibits a low fusion index of 21.9% (Fig. 1f). Thus, it is confirmed that cells cultured in G2 and G3 were more differentiated. Moreover, the results of the BCA assay (Figure S2a) demonstrate that the samples exhibiting high differentiation also exhibit high protein contents. When a myoblast differentiates, proteins such as MyoD, myogenin, troponin T, and MHC are expressed, so that a higher differentiation rate leads to a higher protein content^13^. It is known that serum stimulates the proliferation of myogenic cells, while the withdrawal of serum stimulates differentiation^13–15^. This means that the withdrawal of serum promotes the differentiation of myoblasts in the differentiation stage. However, these are remarkably important experimental results indicating that myoblasts cultured in low serum conditions in the proliferation stage also significantly increase the efficiency of differentiation in the differentiation stage.

### Cost-efficiency evaluation

In general, the use of FBS in the field of cell-based meat causes the high cost of cell-based meat production. Therefore, it is important to reduce the FBS content to present high cost-efficiency^5^. The cost-efficiency of the CPM is evaluated by calculating the cost of the medium consumed when culturing the cells. When manufacturing the CPM, based on a 90-mm dish, three 8-ml portions of the GM are required, giving a sub-total of 24 ml. In addition, two 8-ml portions of DM are required, giving a separate sub-total of 16 ml. The grand total is, therefore, 40 ml of culture medium (GM + DM). The calculation results are presented for the various components of the three media in Table S1.

The cost-efficiency of the CPM is calculated by dividing the protein concentration by the price of the culture medium, as summarized in Table S2. The protein concentration of the CPM was evaluated via the BCA assay. The results provided in Figure S2b indicate that the protein concentration is the highest in G3 (1.3 μg/μl), followed by G2 (1.0 μg/μl) and G1 (0.73 μg/μl). However, since the price of the medium increases in the order of G2 < G1 < G3, the cost-efficiency was calculated as 1.76 for G2, 1.00 for G1, and 0.31 for G3. As a result, given that the most important factor in cell-based meat research is price competitiveness, it is judged that G2 is the most suitable medium for CPM production because it can produce a reasonably large quantity of protein at the lowest price.

### Nutrient contents, flavor, and protein expression

**Fig. 2:**
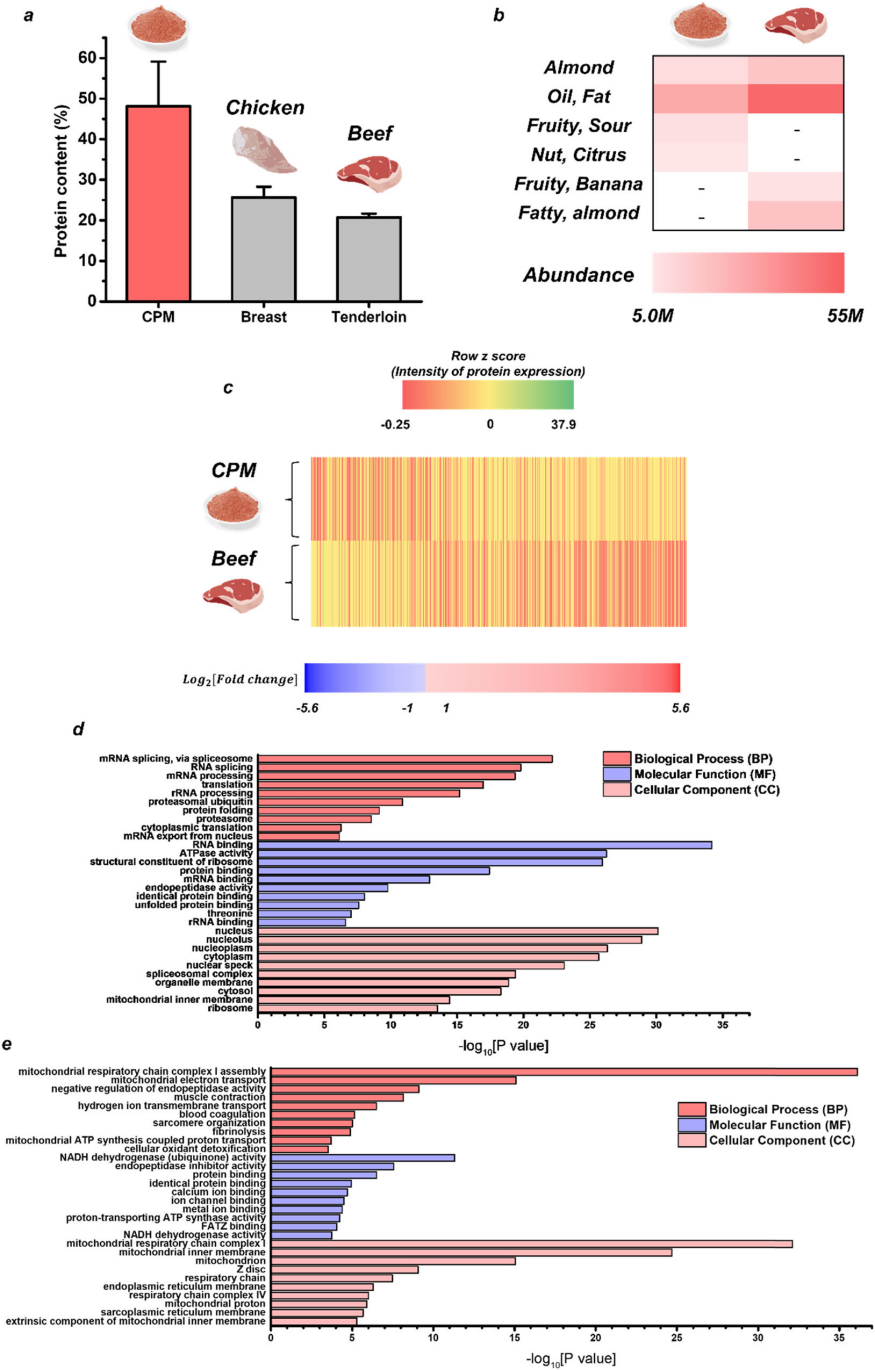
Protein content, flavor, and protein expression of the CPM and beef tissue. **a** a comparison of the protein contents (%) of the CPM (Cell powder meat; G2), chicken breast, and beef tenderloin, and **b** a heat map comparing the flavor intensity of the CPM with that of beef tenderloin. The comparative proteomic analysis of the CPM and bovine muscle tissue: **c** heat maps showing the clustering of differentially expressed proteins in terms of intensity; **d**, **e** gene ontology maps of **d**, the significantly upregulated proteins and **e** the significantly downregulated proteins in the CPM relative to the beef tissue.

As shown in Table S3, an average of 1.6 mg of CPM was obtained per 90-mm dish, and an average of 749.5 μg of protein was obtained from each CPM (G2). Thus, the results presented in Fig. 2a demonstrate that the CPM is a potential food source with a high protein content of 48.1%, which is much higher than that of chicken breast (25.7%) and beef tenderloin (20.7%), which are generally known as high-protein foods.

The flavor analyses were performed on CPM and powdered beef (tenderloin) samples. The results are presented as heat maps in Fig. 2b, where the color intensity is proportional to the peak area of the compound analyzed by GC-MS and are summarized in Table S4. Here, both the CPM and beef contain pentanal, which provides an almond flavor, and hexanal, which is derived from olive oil^16,17^ and provides oily and fatty flavors. Meanwhile, acetic acid, which provides fruity and sour flavors, along with heptanal, which provides fatty, and nutty flavors, are each detected in the CPM, whereas 2-methylbutyl acetate, which provides a fruity flavor, and 3-methylbutanal, which provides fatty and almond flavors, are detected in the beef. These results confirm the potential use of the CPM as a food product with a similar savory flavor to that of meat when grilled.

The proteins of the CPM and bovine tissue are compared by proteome analysis. The CPM is composed of thousands of distinct protein types, with a total of 3047 proteins being measured. Among these, 1050 proteins are expressed at more than twice the intensity of bovine tissue (upregulation), while 491 proteins are expressed at less than half that of bovine tissue (downregulation). This differential expression of protein intensity is represented as a heat map in Fig. 2c, where red indicates the upregulated protein (5.6 > log_2_[fold change] >1) expression and blue indicates the downregulated protein expression (–5.6 < log_2_[fold change] <–1) in the CPM relative to the bovine tissue.

The results of the GO analysis are presented in Fig. 2d, e. From these results, the 10 GO terms with the smallest *p* value (<0.05) are considered the most significantly enriched (upregulation; Table S5, downregulation; Table S6). These upregulated proteins are found to be involved in biological processes (BP) such as RNA splicing, translation, and protein folding, molecular functions (MF) such as RNA binding and protein binding, and in cellular components (CC) such as the nucleus, cytoplasm, membrane, and ribosomes (Fig. 2d). Meanwhile, the downregulated proteins are found to be involved in BPs such as mitochondrial respiratory chain complex 1 assembly, muscle contraction, and sarcomere organization, MFs such as protein binding, and metal ion binding, and CCs such as the mitochondria, Z discs, and sarcomeres. Thus, the proteins constituting the various components of skeletal muscle fibers (mitochondria, sarcomeres, and Z discs) are upregulated in the beef tissue, as are proteins that play roles such as protein binding and muscle contraction. However, because the CPM is not organized up to the level of muscle tissue, proteins that occur at the cellular level, such as those constituting the cell nuclei, cytoplasm, and ribosomes are upregulated. Also, proteins that are known to be expressed in the myoblasts and myotubes, that function in RNA binding, protein binding, and protein folding, are upregulated^18,19^. This suggests that CPM can be manufactured from myoblasts and myotubes, but not from muscle tissue. Nevertheless, the above analysis shows that it can produce a greater amount of protein than beef muscle tissue, along with a similar flavor to that of meat; hence, it can be efficiently manufactured as a potential future food source.

Herein, cell powder meat (CPM) was proposed as a high-protein food source based on the higher differentiation rate of the C2C12 skeletal muscle cell line. It is indicated that myoblasts cultured in low serum conditions at the proliferation stage had a higher fusion index and consequently increased protein content. The CPM was shown to have high nutritional content and a similar flavor to that of meat, thereby demonstrating its potential use in various future food products. It is expected that the as-prepared CPM will become the basis for the mass production of cultured meat. Furthermore, by using fat cells, vascular cells, fibroblasts, and bone cells, CPM with various nutrients, tastes, and flavors can potentially be manufactured. In addition, CPM may be prepared by using the cells of various animals such as cattle, pigs, and chickens. Thus, the present work has demonstrated that the fabrication of CPM can be a groundbreaking strategy for the scaling up and cost reduction of cultured meat.

## Methods

### Cell culture, proliferation, and differentiation

The American Type Culture Collection (ATCC) mouse embryonic myoblast C2C12 cells, as precursors of skeletal muscle cells (passage 8-13) were seeded in a culture dish at a density of 2 *×* 10^3^ cells/cm^2^. The control group(G1) was cultured in Dulbecco’s Modified Eagle’s Medium (DMEM, Gibco® Life Technologies, USA) and 10% (v/v) fetal bovine serum (FBS; WELGENE, Korea) as a standard growth medium (GM) with 1% penicillin/streptomycin antibiotics (PS; Gibco® Life Technologies, USA). The experimental groups (G2 and G3) were cultured in DMEM with 5% FBS (G2) or 5% FBS + 50 μg/ml C -phycocyanin (C-PC, Sigma Aldrich) (G3), each with 1% PS. All three groups were cultured at 37 °C under a humidified atmosphere containing 5% CO_2_ and incubated for 7 days. The GM was replaced every 2 days. Upon reaching 100% confluence (i.e., after 7 days), the culture medium was replaced with a differentiation medium (DM) consisting of 5% horse serum (HS; Thermo Fisher Scientific) and 1% PS, and the cells were differentiated for 5 days. During this time, the DM was replaced every 2 days.

### Preparation of cell powder meat (CPM)

After culturing the C2C12 myoblasts for a total of 12 days according to the procedure described in Section ‘Cell culture, proliferation and differentiation’, the DM was removed and washed with 1*×* phosphate-buffered saline (PBS) solution. The cells were then carefully detached from the dish by using a cell scraper, starting from the edge, and working inwards. The detached cell mass and non-separated cells were collected with distilled water and transferred to a 1.5-ml microtube. Then, centrifugation was performed for 2 min at 300 *×* *g* force to form a pellet, and the supernatant was removed. Finally, after pre-freezing at –20 °C for 12 h, the pellet was lyophilized for 24 h.

### Evaluation of cell proliferation

To evaluate the cell proliferation, C2C12 cells were seeded in a 12-well plate with a density of 2 × 10^3^ cells/cm^2^ in each growth medium (G1, G2, G3), and cultured for 2, 4, and 7 days, with the GM being replaced every 2 days. An assay was performed using a Cell Counting Kit-8 (CCK-8, D-Plus CCK cell viability assay kit, Dongin LS, Korea) in accordance with the manufacturer’s protocol.

### Immunofluorescent staining

The C2C12 cells were cultured in DM for 5 days, then washed twice with 1*×* PBS, fixed in formaldehyde solution (Sigma Aldrich) at room temperature for 15 min, and washed two more times with PBS. To prevent non-specific protein binding, the cells were incubated overnight in a blocking solution consisting of 2% (v/v) bovine serum albumin (BSA), 0.3% (v/v) Triton X-100, and 10% (v/v) horse serum in PBS at 4 °C. Thereafter, the cells were incubated at room temperature for 2 h in myosin heavy chain (MyHC) antibody MF20 that was diluted 100 times in 2% (v/v) BSA and 10% (v/v) HS solution in 1*×* PBS. Then, after washing once with PBS and once with 0.025% (v/v) triton X-100, the secondary antibody, Alexa Flour 594-conjugated Donkey anti-Mouse IgG, was diluted 400 times with the same diluent as above and treated at room temperature for 30 min. After washing once with PBS and once with 0.025% Triton X-100, the fluorescent stain 4′,6-diamidino-2-phenylindole (DAPI) was diluted 250 times with 1% (v/v) BSA solution, and the cell sample was stained for 30 min.

### Fusion assays

The cells that were prepared as described in Section ‘Immunofluorescent staining’ were imaged via confocal microscopy (CLSM; LSM 880, Carl Zeiss) using a *×*10 objective in order to analyze the formation of myotubes by the myoblasts. The cell nuclei were stained blue by the DAPI, and the myotube cells were stained red due to the presence of MyHC and Alexa Flour 594. Hence, the total number of cells in the 850.19 μm *×* 850.19 μm image was indicated by the number of DAPI-stained nuclei, and the number of cells in the myotube was indicated by the number of nuclei in the MyHC-stained area. At least three random fields were captured for each sample, and the average value was used to calculate the fusion index as the number of nuclei in the myotube with two or more nuclei divided by the total number of nuclei, as given by Eq. (1):

1 $$Fusion\;index = \frac{{The\;number\;of\;nuclei\;in\;myotube( \ge 2\;myonuclei)}}{{The\;total\;number\;of\;nuclei}}X\;100$$

### Evaluation of protein content

Before powdering the sample, the detached C2C12 cells were lysed at 4 °C in radioimmunoprecipitation assay (RIPA) lysis buffer (Thermo Fisher Scientific) for 30 min and centrifuged at 10,000 *×* *g* for 10 min to extract the protein from the supernatant. The powdered sample, chicken breast, and beef tenderloin were also lysed in RIPA lysis buffer to extract the protein. The extracted protein was quantified with a bicinchoninic acid assay (BCA) kit (Thermo Fisher Scientific).

### Flavor analysis

To evaluate the potential of the CPM as a food product, samples of the CPM and lyophilized beef (tenderloin) were each grilled on a hot-plate with a little oil at 120 °C for 15 min. The flavor analysis was then performed via the headspace-solid phase microextraction (HS-SPME) method of gas chromatography-mass spectrometry (GC-MS, Agilent 8890 GC system-Agilent 5677B MSD, Agilent Technologies). The analytes were separated on a HP-5ms column (30 m *×* 250 μm *×* 0.25 μm). The temperature of the oven was initially maintained at 40 °C for 5 min then raised to 160 °C at a rate of 4 °C/min, then increased to 250 °C at a rate of 7 °C/min, and maintained for 10 min. The mass spectra (MS) were acquired in normal scanning mode with a source temperature of 230 °C. The volatile compounds were identified by comparison with the data from the spectral library (Agilent Chemstation Integrator). The flavors were referenced from the Flavor Extract Manufacturers Association of the United States (FEMA) and the joint FAO/WHO Expert Committee on Food Additives (JECFA) lists.

### Proteome analysis

Protein samples of CPM and bovine tissue were prepared as described in Section ‘Evaluation of protein content’. Samples were digested in an S-Trap mini spin column (Protifi, USA) and samples were homogenized by 5% SDS in 50 mM TEAB. The samples were heated and alkylated with iodoacetamide at a final concentration of 20 mM. The alkylated proteins were acidified by phosphoric acid, then the protein solution was centrifuged at 4000 *×* *g* for 30 s and washed by a (9 :1, methanol: 50 mM TEAB) solution. The proteins were digested with trypsin gold (Promega) at a 10 (protein) :1 (enzyme) ratio (w/w) and eluted by elution buffers. To compare the proteins of the CPM and bovine tissue, each peptide sample was analyzed using an LC-MS/MS at Korea Basic Science Institute (KBSI). Only proteins that differed more than twice in the intensity of protein expression from CPM and bovine tissue were analyzed, and Gene Ontology (GO) data were obtained using DAVID Bioinformatics Resources (https://david.ncifcrf.gov/home.jsp). GO terms with p-values of < 0.05 were analyzed as significant.

### Statistical analyses

A two-sample *t* test was conducted to determine the statistical validity of the experimental data. Significant differences in mean values between groups were evaluated by statistical analysis, and *p* values of *p < 0.05 and **p < 0.01 were considered significant.

### Reporting summary

Further information on research design is available in the Nature Research Reporting Summary linked to this article.

## Supplementary information

Supplementary Information

Reporting Summary

## Data Availability

The authors declare that the data supporting the findings of this study are available within the paper and supplementary information files. The data also can be available from the corresponding author upon reasonable request.
