# Supplementary material for: High protein-containing new food by cell powder meat
Source: NPJ Sci Food. 2023 Apr 11;7:13. doi: 10.1038/s41538-023-00191-5 (PMC10090064; doi:10.1038/s41538-023-00191-5)
Supplement: Supplementary file 1 — Supplementary Information [file 41538_2023_191_MOESM1_ESM.pdf]

Supplementary Information

High protein-containing new food by cell powder meat

Bumgyu Choi [a], Sohyeon Park [a], Milae Lee [a], Sungwon Jung [a], Hyun Lee [b], Geul Bang [c], Jiyu Kim [a], Heeyoun Hwang [c], Ki Hyun Yoo [d], Dongoh Han [d], Seung Tae Lee [b, e], Won-Gun Koh [a] and Jinkee Hong [a, *]

[a] School of Chemical & Biomolecular Engineering, Yonsei University, 50 Yonsei-ro, Seodaemun-gu, Seoul, 03722, Republic of Korea

[b] Department of Animal Life Science, Kangwon National University, Chuncheon, 24341, Republic of Korea

[c] Research Center for Bioconvergence Analysis, Korea Basic Science Institute, 28119, Republic of Korea

[d] SIMPLE Planet Inc., 48 Achasan-ro 17-gil, Seongdong-gu, Seoul 04799, Republic of Korea

[e] Department of Applied Animal Science, Kangwon National University, Chuncheon, 24341, Republic of Korea

[*] Correspondence to Prof. Jinkee Hong, Ph. D. (E-mail: jinkee.hong@yonsei.ac.kr),

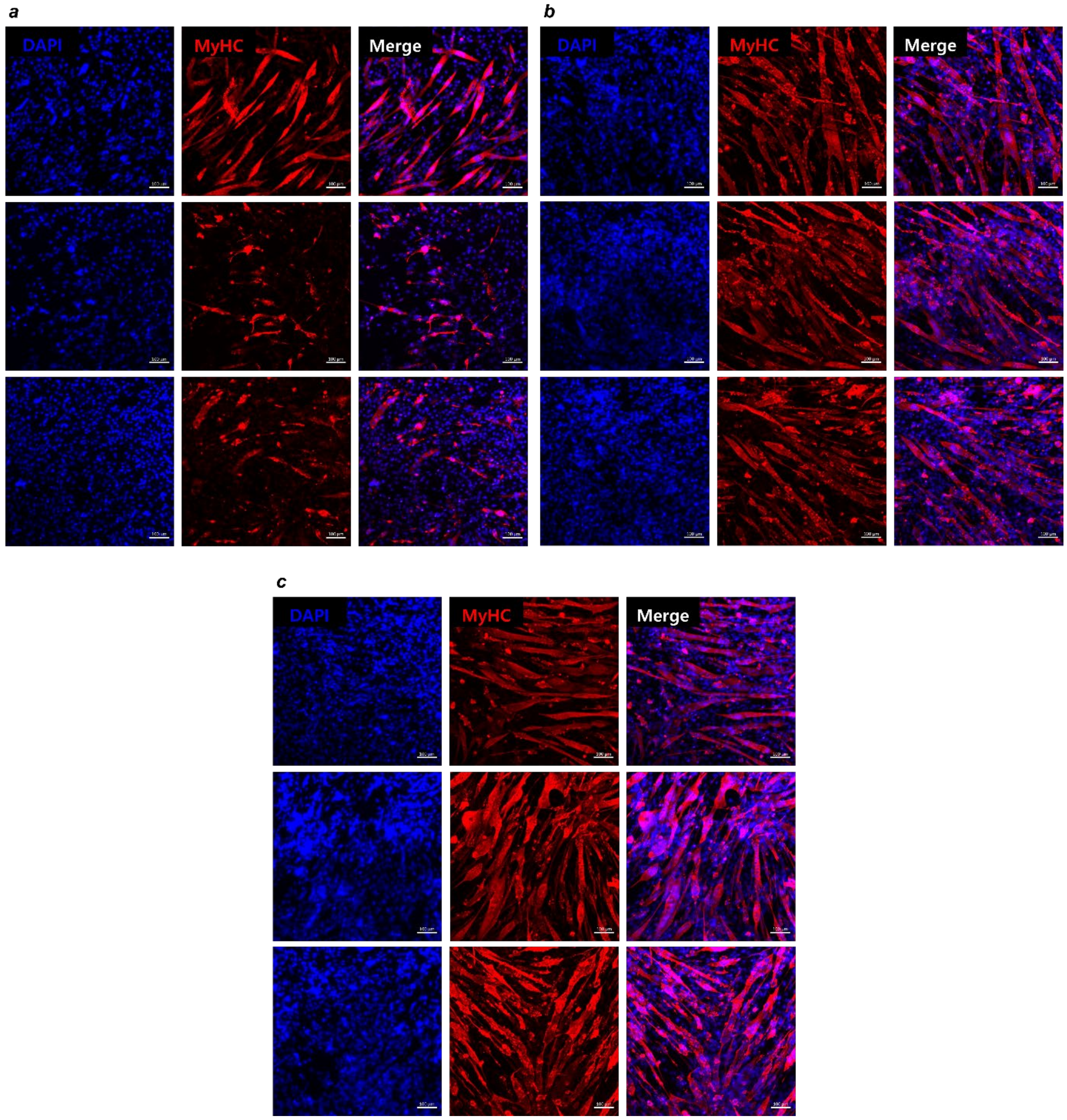

Fig. S1 Confocal images of C2C12 cells stained with DAPI (blue) and MyHC (red) (scale bar: 100 μm) after culturing in various media: (a) G1, (b) G2, and (c) G3 (in each case, n = 3). The three pictures in each condition mean reproducibility.

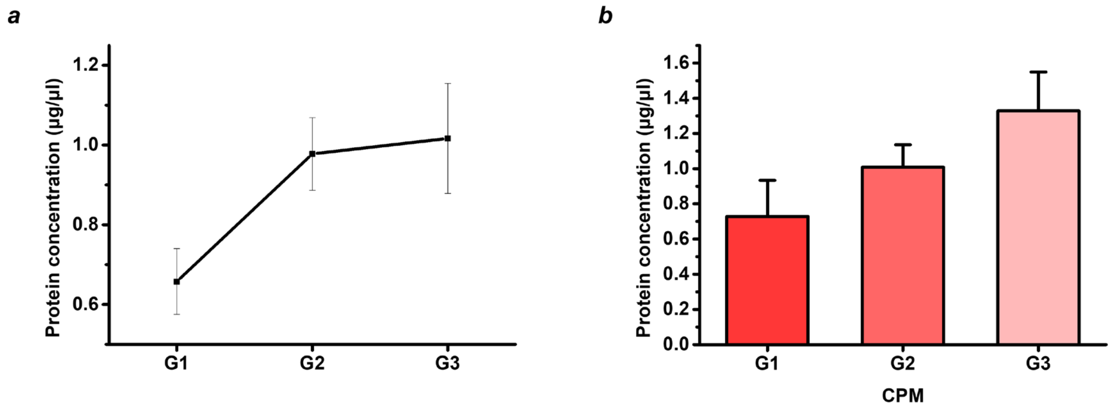

Fig. S2 (a) The protein concentrations of C2C12 pellets after culturing in G1, G2, and G3, and (b) the BCA protein quantification of the corresponding CPMs (data are presented as the mean ± standard deviation; n = 3)

Table S1 Manufacturing cost of the CPM

	G1	G2	G3
DMEM	$2.7/45 ml	$2.9/47.5 ml	$2.9/47.5 ml
FBS	$4.5/5 ml	$2.2/2.5 ml	$2.2/2.5 ml
C-PC	0	0	$50.9/2.5 mg
PS	$0.2/0.5 ml	$0.2/0.5 ml	$0.2/0.5 ml
Growth medium	$7.5/50 ml	$5.4/50 ml	$56.3/50 ml
HS	$0.4/2.5 ml	$0.4/2.5 ml	$0.4/2.5 ml
DM (Horse serum 5%)	$3.6/50 ml	$3.6/50 ml	$3.6/50 ml
Total	$4.7/40 ml	$3.7/40 ml	$28.2/40 ml

The price of each product was calculated based on the price purchased in Korea. As of September 5, 2022, 08:45 UTC, it was calculated as 1KRW=0.00073USD.

Table S2 The cost-efficiencies of the various CPM samples

	G1	G2	G3
Price ($)	4.7	3.7	28.2
Price ratio	1.0	0.8	6.0
Protein concentration (μg/μl)	0.729	1.008	1.329
Protein ratio	1.0	1.4	1.8
Cost-efficiency	1.0	1.76	0.31

Table S3 The weight, the total amount of protein, and protein content of the CPM

CPM (G2) (mg)	Total amount of protein (μg)	Protein content (%)
1.3	788.9	60.7
1.6	697.7	43.6
1.9	762.0	40.1

Table S4 The flavor types and corresponding intensities of the CPM and beef

Compound	CAS no.	Flavor	Peak area (relative)	
			CPM	Beef powder
Pentanal	110-62-3	Almond	9564138	18348431
Hexanal	66-25-1	Oil, fat	29441436	54450145
Acetic acid	64-19-7	Fruit, sour	7845318	–
Heptanal	111-71-7	Citrus, fat, green vegetables, nut	5058060	–
2-methylbutyl acetate	624-41-9	Fruity, apple, banana, pear	–	6791412
3-methylbutanal	590-86-3	Fatty, meaty, almond odor	–	19255768

Table S5 The enriched gene ontology (GO) terms of proteins with significant up-regulation in the CPM relative to the beef tissue.

GO term	Clustering component	Count	P-value	Fold enrichment
mRNA splicing, via spliceosome	Biological process (BP)	51	7.04 E–23	5.250753
RNA splicing		35	1.65 E–20	7.078221
mRNA processing		40	4.43 E–20	5.908776
translation		56	1.10 E–17	3.76013
rRNA processing		31	6.52 E–16	6.053099
proteasomal ubiquitin-independent protein catabolic process		15	1.31 E–11	10.19264
protein folding		27	7.48 E–10	4.169716
proteasome-mediated ubiquitin-dependent protein catabolic process		32	2.92 E–09	3.418914
cytoplasmic translation		19	5.48 E–07	4.085657
mRNA export from nucleus		13	7.53 E–07	5.968662
RNA binding	Molecular function (MF)	124	6.72 E–35	3.470934
ATPase activity		39	5.85 E–27	9.041552
structural constituent of ribosome		73	1.17 E–26	4.359194
protein binding		47	3.72 E–18	4.56246
mRNA binding		40	1.23 E–13	4.042245
endopeptidase activity		18	1.69 E–10	7.094141
identical protein binding		95	9.77 E–09	1.838964
unfolded protein binding		21	2.66 E–08	4.498096
threonine-type endopeptidase activity		9	9.67 E–08	12.66811
rRNA binding		15	2.53 E–07	5.577155
nucleus	Cellular component (CC)	350	7.82 E–31	1.768568
nucleolus		110	1.31 E–29	3.376532
nucleoplasm		221	5.03 E–27	2.073693
cytoplasm		325	2.20 E–26	1.728561
nuclear speck		69	8.63 E–24	4.147499
spliceosomal complex		31	4.13 E–20	8.323049
organelle membrane		24	1.36 E–19	11.50652
cytosol		247	5.16 E–19	1.719811
mitochondrial inner membrane		52	3.66 E–15	3.573697
ribosome		30	2.99 E–14	5.645722

Table S6 The enriched gene ontology (GO) terms of proteins with significant down-regulation in the CPM relative to the beef tissue.

GO term	Clustering component	Count	P-value	Fold enrichment
mitochondrial respiratory chain complex I assembly	Biological process (BP)	34	7.77 E–37	21.64276
mitochondrial electron transport, NADH to ubiquinone		15	8.06 E–16	21.21839
negative regulation of endopeptidase activity		17	7.74 E–10	7.46302
muscle contraction		12	7.03 E–09	10.91232
hydrogen ion transmembrane transport		13	3.23 E–07	6.895977
blood coagulation		10	7.08 E–06	7.344828
sarcomere organization		8	9.57 E–06	10.18483
fibrinolysis		6	1.29 E–05	17.62759
mitochondrial ATP synthesis coupled proton transport		5	1.92 E–04	15.91379
cellular oxidant detoxification		8	2.99 E–04	6.110897
NADH dehydrogenase (ubiquinone) activity	Molecular function (MF)	12	4.90 E–12	20.29091
endopeptidase inhibitor activity		8	2.83 E–08	22.28021
protein binding		19	3.17 E–07	4.431348
identical protein binding		44	1.12 E–05	2.046365
calcium ion binding		32	1.95 E–05	2.341661
ion channel binding		11	3.15 E–05	5.482105
metal ion binding		59	4.05 E–05	1.727509
proton-transporting ATP synthase activity, rotational mechanism		5	5.69 E–05	21.52066
FATZ binding		4	8.99 E–05	37.87636
NADH dehydrogenase activity		4	1.77 E–04	31.56364
mitochondrial respiratory chain complex I	Cellular component (CC)	32	7.77 E–33	20.53954
mitochondrial inner membrane		46	2.06 E–25	7.255435
mitochondrion		64	8.65 E–16	3.156557
Z disc		16	8.51 E–10	8.215816
respiratory chain		8	3.36 E–08	21.74775
endoplasmic reticulum membrane		32	5.11 E–07	2.806161
respiratory chain complex IV		8	9.62 E–07	14.21968
mitochondrial proton-transporting ATP synthase complex		8	1.27 E–06	13.69303
sarcoplasmic reticulum membrane		7	2.09 E–06	17.0262
extrinsic component of mitochondrial inner membrane		6	5.12 E–06	21.32952
